# Supplementary material for: Live imaging of transcription sites using an elongating RNA polymerase II–specific probe
Source: J Cell Biol. 2021 Dec 2;221(2):e202104134. doi: 10.1083/jcb.202104134 (PMC8647360; doi:10.1083/jcb.202104134)
Supplement: Table S1 — lists the reagents and resources used in the study. [file JCB_202104134_TableS1.docx]

Table S1. Reagents and resources

| Reagent or resource | Source | Identifier |
| --- | --- | --- |
| **Chemicals** | | |
| ’’DMEM, high glucose | Nacalai Tesque | Catalog no. 08458-16 |
| FBS | Thermo Fisher Scientific (Gibco) | Catalog no. 10270-106 |
| GPS | Sigma-Aldrich | Catalog no. G1146-100ML |
| FuGENE HD | Promega | Catalog no. E2312 |
| Opti-MEM | Thermo Fisher Scientific | Catalog no. 31985-070 |
| G418 | Nacalai Tesque | Catalog no. 09380-44 |
| Puromycin | InvivoGen | Catalog no. ant-pr |
| FluoroBrite | Thermo Fisher Scientific | Catalog no. A18967-01 |
| Flavopiridol | Sigma-Aldrich | Catalog no. sc-202157 |
| Triptolide | Tocris | Catalog no. 38748-32-2 |
| HaloTag TMR ligand | Promega | Catalog no. KMM-201 |
| Cy3-dUTP | PerkinElmer | Catalog no. NEL578 |
| TRIzol | Thermo Fisher Scientific | Catalog no. 15596026 |
| SuperScript III First-Strand Synthesis System for RT-PCR | Thermo Fisher Scientific | Catalog no. 18080-044 |
| PrimeSTAR | Takara Bio | Catalog no. R010A |
| KOD one PCR Master Mix Blue | Toyobo | Catalog no. KMM-201 |
| Q5 DNA polymerase | New England Biolabs | Catalog no. M0493L |
| PCR purification kit | Qiagen | Catalog no. 28106 |
| In-fusion system | Takara Bio | Catalog no. 634845 |
| BamHI | New England Biolabs | Catalog no. R3136L |
| DpnI | Takara Bio | Catalog no. 1235A |
| EcoRI | Takara Bio | Catalog no. 1166A |
| EcoRI | New England Biolabs | Catalog no. R3101 |
| NotI | Takara Bio | Catalog no. 1166A |
| Lysozyme | Nacalai Tesque | Catalog no. 100940 |
| Proteinase inhibitor cocktail | Nacalai Tesque | Catalog no. 03969-21 |
| Ni-nitrilotriacetic acid agarose | Qiagen | Catalog no. 30210 |
| HiTrap Q column | GE Healthcare | Catalog no. 17-1153-01 |
| Enterokinase Cleavage Capture kit | Novagen | Catalog no. 69067-3 |
| Bradford protein assay | Bio-Rad Laboratories | Catalog no. 500-0006 |
| Blocking One-P | Nacalai Tesque | Catalog no. 05999-84 |
| Tryptone | Nacalai Tesque | Catalog no. 35640-95 |
| Yeast extract | Nacalai Tesque | Catalog no. 36802-16 |
| NaCl | Nacalai Tesque | Catalog no. 31320-05 |
| Glucose | Nacalai Tesque | Catalog no. 16806-25 |
| IPTG | Nacalai Tesque | Catalog no. 19742-94 |
| Tris-hydroxymethyl-aminomethane | Fujifilm Wako Pure Chemical | Catalog no. 204-07885 |
| Glycerol | Fujifilm Wako Pure Chemical | Catalog no. 075-00616 |
| Imidazole | Fujifilm Wako Pure Chemical | Catalog no. 322-91221 |
| SDS-polyacrylamide gel | Fujifilm Wako Pure Chemical | Catalog no. 198-15041 |
| BSA | Fujifilm Wako Pure Chemical | Catalog no. 2320 |
| PBS | Fujifilm Wako Pure Chemical | Catalog no. 048-29805 |
| o-Phenylenediamine solution | Fujifilm Wako Pure Chemical | Catalog no. 158-01671 |
| HCl | Nacalai Tesque | Catalog no. 18321-05 |
| **Special plastic and glass ware** | | |
| 35-mm Glass-bottomed dishes | AGC Technology Solutions | Catalog no. 3971-035 |
| 20-ml Open column (Poly-Prep Chromatography Columns) | Bio-Rad Laboratories | Catalog no. 9704652 |
| Microtiter ELISA plates | Greiner Bio-One | Catalog no. 655061 |
| **Recombinant DNA** | | |
| Super PiggyBac Transposase Expression Vector | System Biosciences | Catalog no. PB210PA-1 |
| PB-EF1α-MCS-IRES-Neo PiggyBac cDNA Cloning and Expression Vector | System Biosciences | Catalog no. PB533A-2 |
| PB-CMV-MCS-EF1α-Puro PiggyBac cDNA Cloning and Expression Vector | System Biosciences | Catalog no. PB510B-1 |
| pTrcHis A, B, and C bacterial expression vectors | Thermo Fisher Scientific | Catalog no. V36020 |
| Halo-BRD4 expression vector | Promega | ORF Clone pFN21AE9668; catalog no. FHC11882 |
| Halo-p300 expression vector | Promega | EP300 ORF Clone pFN21AB4967; catalog no. FHC01787 |
| sfGFP-N1 vector | Addgene | Addgene plasmid 54737 |
| SF2(SRSF1/ASF)-mRFP1 expression vector | Yomoda et al., 2008 | NA |
| mCherry-PCNA expression vector | Imada et al., 2021 | NA |
| H2B-Halo expression vector | This study | BamHI/NotI fragment of H2B was cloned into BamHI/NotI-digested PB533-based HaloTag vector |
| Halo-PCNA expression vector | Sato and Kimura, 2021 | NA |
| **Oligonucleotide primers (5′ to 3′)** | | |
| 42B3 scFv VH_s | This study (cloning 42B3 VH) | 5′-CGAATTCGCCATGGCCGAAGTGCAGCTGGTGGAGTC-3′ |
| 42B3 scFv VH_as | This study (cloning 42B3 VH) | 5′-TGAACCGCCTCCACCTGAGGAGACGGTGACCGTGG-3′ |
| 42B3 scFv VL_s | This study (cloning 42B3 VL) | 5′-TCTGGCGGTGGCGGATCGCAGGCTGTTGTGACTCAGGAA-3′ |
| 42B3 scFv VL_as | This study (cloning 42B3 VL) | 5′-TGGATCCGCCTTGGGCTGGCCTAGGACAGTC-3′ |
| LINK primer1 | This study (connecting VH and VL) | 5′-GTCTCCTCAGGTGGAGGCGGTTCAGGCGGAGGTGGCTCTGGCGGTGGCGGATCG-3′ |
| LINK primer2 | This study (connecting VH and VL) | 5′-CGATCCGCCACCGCCAGAGCCACCTCCGCCTGAACCGCCTCCACCTGAGGAGAC-3′ |
| scFv primer_s | This study (subcloning) | 5′-CTCGAGCTCAAGCTTCGAATTCGCCATGGCCGAAG-3′ |
| scFv primer_as | This study (subcloning) | 5′-CATGGTGGCGACCGGTGGATCCGCCTTGGGC-3′ |
| R78K/A80T_s | This study (making point mutation) | 5′-CAGGTACAGGGTGTTCTTGGCATTGTCTCTGGAGATG-3′ |
| R78K/A80T_as | This study (making point mutation) | 5′-CATCTCCAGAGACAATGCCAAGAACACCCTGTACCTG-3′ |
| A108Q_s | This study (making point mutation) | 5′-GACCGTGGTCCCTTGGCCCCAGACATCG-3′ |
| A108Q_as | This study (making point mutation) | 5′-CGATGTCTGGGGCCAAGGGACCACGGTC-3′ |
| M95I_s | This study (making point mutation) | 5′-CTGAGGACACGGCCATCTATTACTGTGCAAGAG-3′ |
| M95I_as | This study (making point mutation) | 5′-CTCTTGCACAGTAATAGATGGCCGTGTCCTCAG-3′ |
| M95V_s | This study (making point mutation) | 5′-GTCTGAGGACACGGCCGTCTATTACTGTGCAAG-3′ |
| M95V_as | This study (making point mutation) | 5′-CTTGCACAGTAATAGACGGCCGTGTCCTCAGAC-3′ |
| V112L_s | This study (making point mutation) | 5′-CCAAGGGACCACGCTCACCGTCTCCTC-3′ |
| V112L_as | This study (making point mutation) | 5′-GAGGAGACGGTGAGCGTGGTCCCTTGG-3′ |
| RPB3-Halo_s | This study (cDNA cloning) | 5′-AGAGCTAGCGAATTCGCCATGCCGTACGCCAACC-3′ |
| RPB3-Halo_as | This study (cDNA cloning) | 5′-GGCGATGGGATCCGCATTTATGGTTAGCACATCACTCTG-3′ |
| CDK9-Halo_s | This study (cDNA cloning) | 5′-TAGAGCTAGCGAATTCGCCATGGCAAAGCAGTACG-3′ |
| CDK9-Halo_as | This study (cDNA cloning) | 5′-GGTGGCGATGGGATCCGCGAAGACGCGCTCAAACTCC-3′ |
| CDK12-Halo_s | This study (cDNA cloning) | 5′-TAGAGCTAGCGAATTCGCCATGCCCAATTCAGAGAG-3′ |
| CDK12-Halo_as | This study (cDNA cloning) | 5′-GGTGGCGATGGGATCCGCGTAAGGAACTCCTCTCCC-3′ |
| LEO1-Halo_s | This study (cDNA cloning) | 5′-TAGAGCTAGCGAATTCGCCATGGCGGATATGGAGG-3′ |
| LEO1-Halo_as | This study (cDNA cloning) | 5′-GGTGGCGATGGGATCCGCATCATCATCTTCTTCCTCTTC-3′ |
| **Cells** | | |
| HeLa | Obtained from Peter R. Cook  (Oxford University, Oxford, UK) | NA |
| **Antibodies** | | |
| Mouse IgG for Ser2ph | Stasevich et al., 2014 | CMA602; RRID: AB_2819246 |
| Mouse IgG for Ser5ph | Stasevich et al., 2014 | CMA603; RRID: AB_2827955 |
| Mouse IgG for Ser2ph | Mab Institute Inc. | 42B3 |
| Anti-GFP pAb-HRP-DirecT | MBL | Catalog no. 598-7 |
| Peroxidase-conjugated donkey antimouse IgG | Jackson ImmunoResearch | Catalog no. 715-035-150 |
| **Instruments** | | |
| Chromatography system | GE Healthcare | AKTA prime plus |
| Plate reader | Thermo Fisher Scientific | Varioskan |
| Spectrophotometer | Thermo Fisher Scientific | NanoDrop |
| Spinning disk high-resolution confocal microscopy system | Olympus | IXplore SpinSR |
| Scanning confocal microscopy system | Olympus | FluoView FV1000 |
| HILO microscopy system | Custom built | Based on Olympus CellTIRF |
| Heated stage | Tokai Hit | INU-WSKMOR-F0-FP, STXG-IX3WX-SET |
| CO2 control system | Tokken | MIGM/OL |
| Sonicator | Branson | Sonifier250 |
| **Software and algorithms** | | |
| ImageJ Fiji 1.52d | National Institutes of He[alth](C:\\Users\\vingerson\\Documents\\_WORK_Documents\\JCB\\_Copy-editing\\202104134\\PER\\althhttps:\\i) | [https://i](C:\\Users\\vingerson\\Documents\\_WORK_Documents\\JCB\\_Copy-editing\\202104134\\PER\\althhttps:\\i)magej.net/Fiji |
| NIS Elements version 5.21 | Nikon | NA |
| MATLAB version 9.10.0.1739362 | MathWorks | NA |
| BoxPlotR | Tyers and Rappsilber laborato[ries](C:\\Users\\vingerson\\Documents\\_WORK_Documents\\JCB\\_Copy-editing\\202104134\\PER\\rieshttp:\\) | [http://](C:\\Users\\vingerson\\Documents\\_WORK_Documents\\JCB\\_Copy-editing\\202104134\\PER\\rieshttp:\\)shiny.chemgrid.org/boxplotr/ |
| Excel 2019 | Microsoft | NA |
