# Supplementary figures and images for: Live imaging of transcription sites using an elongating RNA polymerase II–specific probe
Source: J Cell Biol. 2021 Dec 2;221(2):e202104134. doi: 10.1083/jcb.202104134 (PMC8647360; doi:10.1083/jcb.202104134)

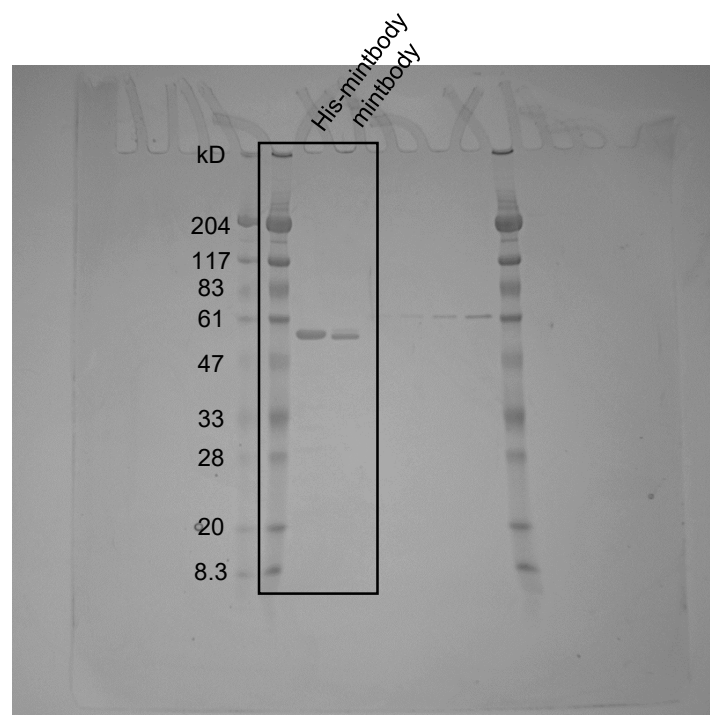

Supplement: SourceData F4 — contains original blots for Fig. 4. [file JCB_202104134_SourceDataF4.pdf]
